# Association between acute kidney injury and delirium in critically ill patients: a retrospective cohort study using two independent databases

**DOI:** 10.1186/s13054-026-05922-0

**Published:** 2026-03-05

**Authors:** Christian Porschen, Christian Strauß, Sean M. Bagshaw, John A. Kellum, Sven G. Meuth, Paul Brauckmann, Ngoc-Anh Thi Nguyen, Michael Fujarski, Keyvan Mahjoory, Carla Barbara Schwienhorst, Alexander Zarbock

**Affiliations:** 1 Department of Anesthesiology, Intensive Care, and Pain Medicine, University Hospital Muenster, University of Muenster, 48149 Muenster, Germany; 2 Department of Critical Care Medicine, Faculty of Medicine and Dentistry, University of Alberta and Alberta Health Services, Edmonton, AB Canada; 3 Center for Critical Care Nephrology, Department of Critical Care Medicine, University of Pittsburgh, Pittsburgh, PA USA; 4 Department of Neurology, Medical Faculty, Heinrich-Heine University Düsseldorf, Düsseldorf, Germany; 5 Institute for Medical Informatics, University of Muenster, Muenster, Germany; 6 Institute of Translational Psychiatry, University of Muenster, Muenster, Germany; 7 Institute of Mathematics, University of Muenster, Muenster, Germany

## Abstract

**Background:**

Acute kidney injury (AKI) and delirium are common complications in critically ill patients, both associated with adverse outcomes. Evidence suggests AKI may induce neuroinflammation and impair clearance of neurotoxic metabolites, but the clinical relationship between AKI and delirium remains incompletely characterized. We hypothesized that AKI increases the risk of delirium in a biological gradient.

**Methods:**

We conducted a retrospective cohort study using two independent databases: MIMIC-IV (*n* = 15,219 patients) and a local institutional database (Reality) from University Hospital Münster (*n* = 3,461 patients). Adult ICU patients with length of stay > 12 h and validated delirium monitoring using the Confusion Assessment Method for the ICU (CAM-ICU) were included, with a positive assessment defining delirium presence. Patients with neurological/neurosurgical primary diagnoses or pre-existing cognitive impairment, as well as patient with delirium onset before AKI were excluded. AKI was classified according to KDIGO criteria. We employed landmark analysis at 24 h post-ICU admission where AKI-status was recorded as the exposure variable. Subsequent delirium was recorded as follow-up during the ICU stay. Propensity score matching, and a Fine-Gray Model were added to assess the time-based association between AKI and subsequent delirium, independently of illness severity.

**Results:**

At the 24-hour landmark, AKI was present in 58.6% (MIMIC-IV) and 55.6% (Reality) of patients. Rates of subsequent delirium were significantly higher in patients with AKI compared to those without: 25.6% versus 15.7% in MIMIC-IV (OR 1.84, CI 1.68–2.04, *p* < 0.001) and 12.1% versus 6.7% in Reality (OR 1.95, CI 1.44–2.64, *p* < 0.001). After adjustment for confounders, AKI remained associated with delirium (adjusted-odds ratio [OR] 1.16, 95% CI 1.10–1.23 in MIMIC-IV; OR 1.20, 95% CI 1.01–1.41 in Reality). A biological gradient was observed, with delirium rates increasing progressively across AKI stages. In MIMIC-IV, adjusted-OR increased from 1.42 for Stage 1 to 4.11 for Stage 3 AKI (trend OR 1.55 per stage, *p* < 0.001). The Reality cohort showed similar patterns (Stage 1 OR 1.01 and Stage 3 OR 1.92, trend OR 1.25 per stage, *p* < 0.01). Propensity score matching confirmed these findings.

**Conclusion:**

Acute kidney injury was associated with a higher risk of subsequent delirium in critically ill patients, with a graded association across AKI severity stages. Prospective studies are needed to determine whether interventions targeting AKI prevention or treatment can also reduce delirium burden.

**Supplementary Information:**

The online version contains supplementary material available at 10.1186/s13054-026-05922-0.

## Introduction

During critical illness, different organs are injured, including the brain and kidneys. Acute kidney injury (AKI), which occurs in up to 50% of critically ill patients [1], is associated with increased short- and long term morbidity and mortality [2–7]. Acute brain dysfunction, which can manifest as delirium, is another common organ dysfunction in critically ill patients that is also associated with adverse short- and long-term outcomes. In survivors of critical illness, delirium is also associated with long-term cognitive impairment [8–15]. 

AKI is a systemic disease that is known to affect other organs and may subsequently contribute to poor outcomes through adverse effects on distant organs [8, 16–23]. Recent studies have shown that AKI can induce inflammation in different remote organs, including the brain [20, 22]. Another mechanism that might contribute to brain dysfunction during AKI is that the reduced kidney function might reduce the clearance of metabolites, medications or other potential neurotoxins [24].

To date, few studies have investigated the association between AKI and delirium in critically ill patients [23, 25–28]. Surrogate markers of AKI, including serum urea, have been identified as risk factors for the development of delirium [29, 30]. In addition, secondary analysis of clinical trials and prospective cohort studies have suggested that early initiation of renal replacement therapy (RRT) may modify the risk of development of delirium and persistent coma [23, 31]. While these studies provided valuable insights, certain limitations should be acknowledged. These include the use of a single threshold to define kidney dysfunction, limited exploration of the temporal relationship between delirium and AKI, and the absence of differentiation between acute and chronic kidney disease.

We hypothesized that AKI during critical illness increases the risk for developing subsequent delirium in the intensive care unit (ICU). We also hypothesized that the severity of AKI would modify the occurrence of delirium. To test these hypotheses, we examined the relationship between AKI and delirium in two large, independent patient cohorts by establishing a landmark analysis with AKI as the exposure at different time points and the subsequent occurrence of delirium as the outcome. To further investigate the mechanistic pathways by which AKI can contribute to delirium we also established landmark analysis were low glomerular filtration rate (GFR) as well as fluid balance were used as categorical exposure variables with delirium as the outcome.

## Materials and methods

### Study design and setting

We conducted a retrospective cohort study to investigate the association between AKI and delirium in critically ill patients. The study utilized two independent cohorts: the MIMIC-IV database Version 2.2 and a local institutional database (Reality). The MIMIC-IV database contains de-identified health data from patients admitted to intensive care units at Beth Israel Deaconess Medical Center between 2008 and 2022 [32, 33]. The Reality cohort comprised patients admitted to intensive care units at the University Hospital Muenster between 2001 and 2021. The use of both databases was approved by the respective institutional review boards, with waiver of informed consent due to the retrospective nature and use of de-identified data.

We included adult patients (≥ 18 years) with their first ICU admission during the study period. Patients were required to have an ICU length of stay exceeding 12 h and at least one Confusion Assessment Method-ICU (CAM-ICU) [34] applied every four days to ensure adequate delirium monitoring. We excluded patients admitted to neurological ICUs, patients after neurosurgical procedures, trauma patients with major head injury and patients with pre-existing neurological or psychiatric conditions such as any form of dementia including Alzheimer’s disease or schizophrenia. Patients with any type of intracranial bleedings or stroke were also excluded. Time points where patients were scored − 4 or -5 on the Richmond Agitation-Sedation Scale (RASS) based on the use of sedatives were not included in the analysis as CAM-ICU could not be assessed and such patients were not classified with regards to the out-come. The created cohort was searched for episodes of AKI during their ICU stay, based on the KDIGO criteria, using the pyAKI software package version 1.0, using urine output, serum creatinine and status of RRT [35]. Baseline creatinine was defined as the lowest available serum creatinine value prior to ICU admission. This approach was chosen to minimize misclassification of AKI in the absence of reliable outpatient data. In the Reality and MIMIC cohorts, urine output was documented as hourly totals and mapped to KDIGO urine output criteria accordingly. Need for renal replacement therapy was classified as stage 3 AKI as stated in the KDIGO Guidelines. AKI exposure at the 24 h time point was evaluated for the landmark analysis.

The primary outcome was the incidence of delirium, defined as the first occurrence of a positive CAM-ICU assessment during the ICU stay as a dichotomous outcome. Delirium was assessed using the CAM-ICU, a screening tool designed for critically ill patients, including those receiving mechanical ventilation. The CAM-ICU evaluates four key features: (1) acute onset or fluctuating course of mental status changes, (2) inattention, (3) altered level of consciousness and (4) disorganized thinking. A positive CAM-ICU assessment requires the presence of features 1 and 2, plus either feature 3 or 4. The tool demonstrates high sensitivity (95–100%) and specificity (89–93%) for delirium detection in ICU populations [34]. In both cohorts, delirium was defined as the first occurrence of a positive CAM-ICU assessment during the ICU stay. The requirement of at least one CAM-ICU assessment every four days was applied as a minimum data completeness criterion. To better characterize delirium assessment frequency, we quantified the number of CAM-ICU assessments per patient and per ICU day in each cohort.

### Statistical methods

We identified potential confounders based on clinical knowledge, data availability and previous literature. Baseline confounders included age, weight, and clinical severity scores measured within the first 24 hours of ICU admission to avoid including variables that might be consequences of AKI rather than true confounders. Further confounders included baseline serum creatinine and lactate levels. Clinical severity was assessed using the Sequential Organ Failure Assessment (SOFA) score and Simplified Acute Physiology Score II (SAPS II). To account for sedative exposure as a potential confounder, we additionally included cumulative doses of commonly used sedatives administered from ICU admission until the landmark timepoint. These included propofol, dexmedetomidine, midazolam and clonidine, each entered as separate covariates in the adjusted models. In the Reality cohort, we additionally collected information on comorbidities and medication exposures that might influence delirium or AKI risk. Unfortunately, this data was not available for MIMIC-IV. To avoid introducing over-granularity in the confounders, antihypertensive medications and oral antidiabetics were grouped together for further analysis. Missing covariate data were handled using multiple imputation by chained Eq. [36] A directed acyclic graph illustrating the assumed causal relationships between AKI, delirium, illness severity, and sedation is provided in the Supplements, under Figure S7. To examine the role of sedative exposure in the association between acute kidney injury (AKI) and delirium, we extracted cumulative doses of commonly used ICU sedatives and analgesics up to the landmark time and included them as covariates in landmark-based regression models (figures S5 and S6). Continuous medication variables were standardized prior to analysis. We fitted sequential logistic models including (1) AKI alone, (2) AKI adjusted for baseline demo-graphic and illness severity variables, and (3) AKI additionally adjusted for sedative exposure. Complementary models including sedatives alone and sedatives jointly with AKI, as well as stratified analyses by AKI status, were performed to assess whether sedatives acted as confounders or potential intermediates in the AKI–delirium association.”

### Statistical analysis

Descriptive statistics were presented as means with standard deviations for continuous variables and frequencies with percentages for categorical variables. We compared delirium proportions between AKI and non-AKI groups using chi-square tests and calculated absolute risk differences. To assess the association between AKI and delirium while controlling for confounding, as well as time-dependent bias, we employed multiple analytical approaches.

As our primary analysis, we performed a landmark analysis using a 24-hour post-ICU admission timepoint. Patients were required to survive beyond the landmark time without developing delirium to be eligible for analysis. AKI exposure status was determined at the landmark timepoint, and patients were subsequently followed from the landmark forward to assess incident delirium. For the landmark analysis, we used multivariable logistic regression to estimate odds ratios. To further assess the influence of CAM-ICU documentation on the results, a sensitivity analysis restricted to only patients with daily CAM-ICU assessments across their ICU-stay was performed. Kaplan-Meier survival curves were constructed to visualize the time-to-delirium.

To further address potential confounding within the landmark analysis, we conducted a propensity score matching analysis. Propensity scores for AKI exposure at the landmark timepoint were estimated using logistic regression, including all baseline confounders. AKI patients were matched 1:1 to non-AKI patients using nearest neighbor matching with replacement. The balance of matched pairs was assessed using McNemar’s test for the primary outcome.

Lastly, to evaluate for a biological gradient, we analyzed the association between AKI severity stages and delirium risk. We created indicator variables for each AKI stage (1, 2, and 3) with no AKI as the reference category. Additionally, we treated AKI severity as an ordinal variable (0, 1, 2, 3) to test for linear trend. Kaplan-Meier survival curves were constructed to visualize time-to-delirium by AKI severity, with log-rank tests used to assess statistical significance.

We performed sensitivity analyses using landmark timepoints at 48-, 72-, and 96-hours post-ICU admission to assess the temporal relationship between AKI timing and effect magnitude. As an additional sensitivity analysis, we investigated alternative exposure definitions. Therefore, we also established additional exposure categories at the 24 h landmark with GFR as the defining parameter. For GFR, a cutoff of 60 ml/min/1,73 m^2^ was used with patients either falling in the “normal” or “low” GFR group.

As a complementary approach, we performed Fine–Gray subdistribution hazards models to account for death during the ICU stay as a competing risk for delirium. AKI status and covariates were defined at the prespecified landmark time point and treated as fixed exposures. This approach estimates the association between AKI and the cumulative incidence of delirium while appropriately accounting for the competing risk of death. All statistical analyses were performed using Python 3.12 with pandas, statsmodels, scikit-learn, and lifelines packages. Statistical significance was set at *p* < 0.05, and all tests were two-sided. Landmark analyses were used to enforce temporal ordering between AKI exposure and subsequent delirium and represent the primary analysis. Time-updated models and competing-risk analyses were employed as complementary approaches to assess robustness across different analytical frameworks.

## Results

### Patient flow and baseline characteristics

The MIMIC-IV cohort included 15,219 patients admitted to ICUs while the Reality cohort comprised of 3,461 patients after applying exclusion criteria (Figure S1). For our primary 24-hour landmark analysis, 10,818 (71.1%) patients in the MIMIC cohort met eligibility criteria after excluding patients with an ICU stay shorter than 24 h (*n* = 1,252) or early delirium occurrence within 24 h (*n* = 3,148). The Reality cohort comprised 3,461 patients with sufficient data, with 2,213 (63.9%) patients meeting eligibility criteria after applying identical exclusion criteria for our primary analysis. Baseline characteristics for both full cohorts are presented in supplemental Tables 1–3. Median time to first positive delirium screen was 2.5 days in the Reality cohort and 1.16 days in the MIMIC cohort. Average CAM-ICU assessments per patient day in the ICU in the MIMIC cohort was 0.76 with a STD of 0.27 and 0.50 with a STD of 0.18 for the Reality cohort. Stratified by AKI status, average CAM-ICU assessments per patient day in the ICU in the MIMIC cohort was 0.76 (STD 0.28) for no AKI, 0.76 (STD 0.26) for AKI Stage 1, 0.79 (STD 0.26) for AKI Stage 2 and 0.76 (STD 0.26) for AKI Stage 3. In the Reality cohort it was 0.52 (STD 0.17) for no AKI, 0.48 (STD 0.18) for AKI Stage 1, 0.47 (STD 0.13) for AKI Stage 2 and 0.46 (STD 0.13) for AKI Stage 3.

### Landmark analysis

**Fig. 1:**
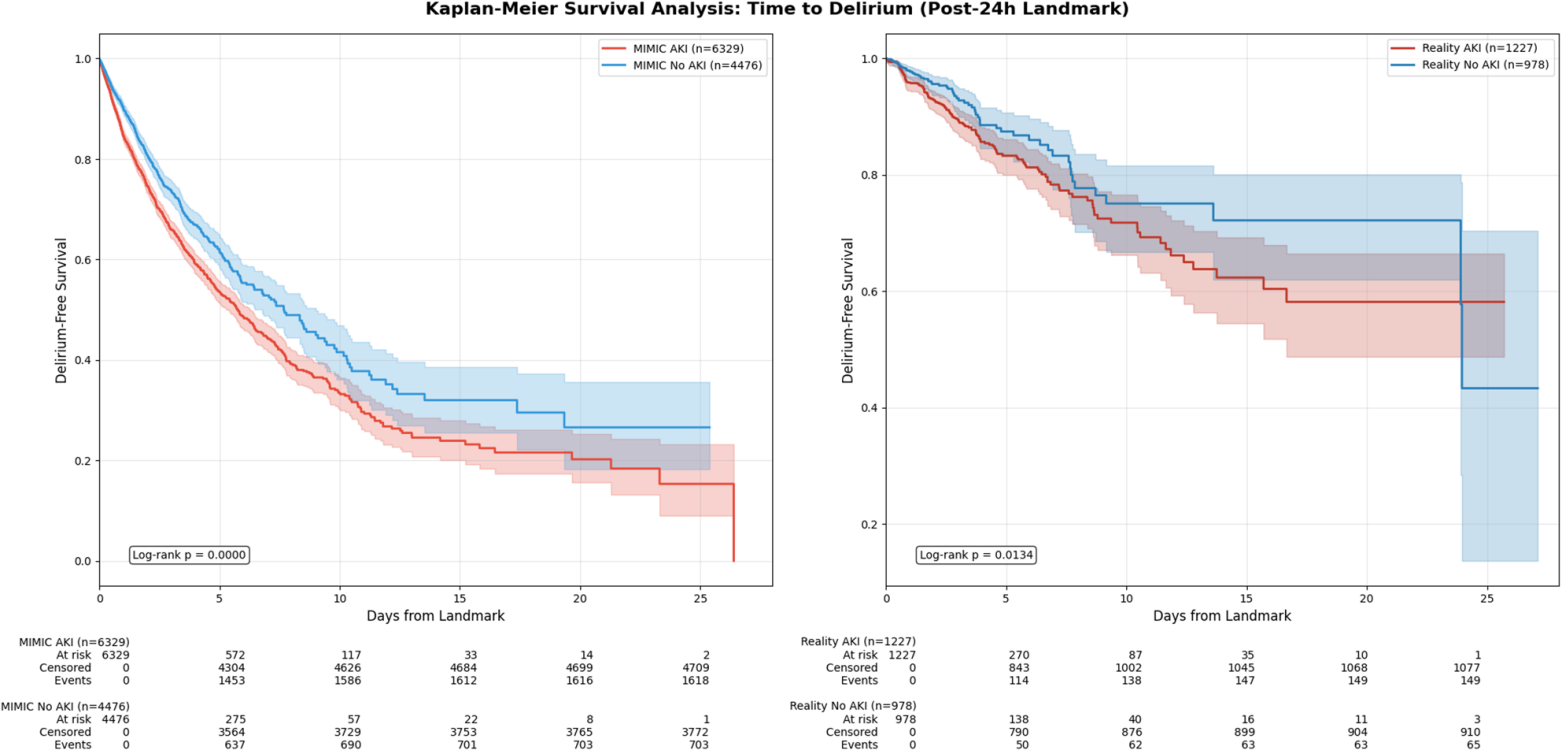
Kaplan-Meier Survival Curves for Time to delirium by AKI Status at 24-Hour Landmark: Kaplan-Meier curves showing delirium-free survival from the 24-hour landmark timepoint in the MIMIC-IV discovery cohort (left panel) and Reality validation cohort (right panel). Blue curves represent patients without AKI and red curves represent patients with AKI at the landmark timepoint. Shaded areas indicate 95% confidence intervals. AKI, acute kidney injury

At the 24-hour landmark timepoint, AKI was present in 6,336 (58.6%) patients in MIMIC-IV and 1230 patients (55.6%) in the Reality cohort. Post-landmark delirium occurred in 2,325 (21.5%) patients in MIMIC-IV and 215 (9.7%) in the Reality cohort. Crude delirium rates were significantly higher in patients with AKI at the 24-hour landmark compared to those without AKI in both cohorts (Table 1, Figures S2 and S3). In the MIMIC-IV cohort, 1,621 of 6,336 patients (25.6%) with AKI developed delirium, compared to 704 of 4,482 patients (15.7%) without AKI, representing an unadjusted OR of 1.85 (CI: 1.68–2.04, *p* < 0.001). This association was confirmed in the Reality cohort, where delirium occurred in 149 of 1,230 patients (12.1%) with AKI versus 66 of 983 patients (6.7%) without AKI, OR 1.95 (CI: 1.44–2.64, *p* < 0.001). In adjusted analyses, AKI remained significantly associated with increased delirium risk in both cohorts. In MIMIC-IV, the adjusted OR was 1.16 (95% CI 1.10–1.23, *p* < 0.01). In the Reality cohort, the association was also significant (adjusted OR 1.20, 95% CI 1.01–1.41, *p* = 0.03). Kaplan-Meier-Curves for both are shown in Fig. 1.

**Table 1:** Crude Delirium Rates sorted by AKI Status at 24-Hour Landmark: Unadjusted delirium rates in patients with and without acute kidney injury (AKI) at the 24-hour landmark timepoint in both cohorts

Cohort	No AKI	AKI present	Absolute risk difference (CI)	*P*-value (AKI vs. No-AKI)
MIMIC-IV	704/4,482 (15.7%)	1,621/6,336 (25.6%)	9.9% (8.36–11.39)	< 0.001
Reality	65/983 (6.7%)	149/1,230 (12.1%)	5.4% (3.11–7.90)	< 0.001

Values are presented as number of events/total number of patients (percentage). Risk differences including 95% confidence intervals and p-values from chi-square tests are shown

Across both cohorts, AKI at the landmark was associated with increased delirium risk in unadjusted models, with progressive attenuation after adjustment for baseline severity and sedative exposure. However, AKI remained independently associated with delirium after full adjustment. Sedative-focused analyses demonstrated strong associations between cumulative exposure to propofol, midazolam, and lorazepam and delirium, while dexmedetomidine showed weaker and inconsistent effects. In models including both sedatives and AKI, AKI retained a significant association with delirium, indicating that sedative exposure explained part, but not all, of the observed relationship. A complete overview of significance levels and detailed ORs can be found in the Supplements under Table S5.

### Association between AKI severity and delirium rate

**Fig. 2:**
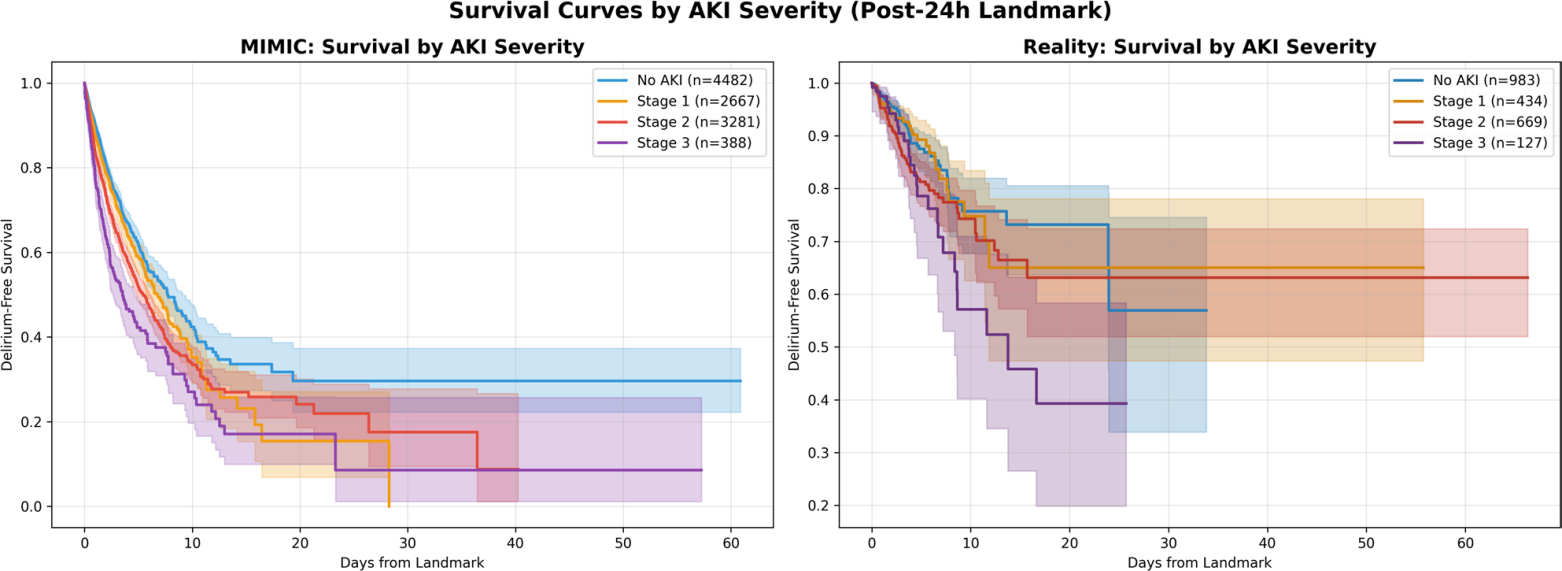
Kaplan-Meier Survival Curves by AKI Severity at 24-Hour Landmark: Kaplan-Meier curves showing delirium-free survival from the 24-hour landmark timepoint stratified by AKI severity in the MIMIC-IV discovery cohort (left panel) and Reality validation cohort (right panel). Curves represent patients with no AKI (blue), AKI Stage 1 (green), AKI Stage 2 (orange), and AKI Stage 3 (red). Shaded areas indicate 95% confidence intervals. Both cohorts demonstrate a biological gradient with progressively lower delirium-free survival across increasing AKI severity stages. AKI, acute kidney injury

AKI severity distribution differed between cohorts. In MIMIC-IV, AKI-Stages were distributed as: Stage 1 (*n* = 2,758, 25.5%), Stage 2 (*n* = 3,974, 36.7%), and Stage 3 (*n* = 1,174, 10.9%). The Reality cohort showed: Stage 1 (*n* = 247, 17.2%), Stage 2 (*n* = 791, 55.0%), and Stage 3 (*n* = 162, 11.3%). Both cohorts demonstrated association between increasing AKI severity and delirium rate (Fig. 2; Table 2). In the MIMIC-IV cohort, delirium rates increased progressively across AKI stages: 343 of 2,912 patients (11.8%) without AKI, 477 of 2,758 patients (17.3%) with Stage 1 AKI, 926 of 3,974 patients (23.3%) with Stage 2 AKI, and 579 of 1,174 patients (49.3%) with Stage 3 AKI. After adjustment for baseline confounders, the odds ratios demonstrated a stepwise increase: Stage 1 (OR 1.42, 95% CI 1.21–1.65, *p* < 0.001), Stage 2 (OR 2.11, 95% CI 1.83–2.43, *p* < 0.001), and Stage 3 (OR 4.11, 95% CI 3.40–4.97, *p* < 0.001). The trend test confirmed a significant linear relationship (OR 1.55 per stage increase, 95% CI 1.46–1.64, *p* < 0.001).

**Table 2:** Biological Gradient Between AKI Severity and Delirium Risk at 24-Hour Landmark: Delirium rates and adjusted odds ratios by AKI severity stage in both cohorts at the 24-hour landmark timepoint

AKI Stage	MIMIC-IV delirium rate	Adjusted OR (95% CI)	Reality delirium rate	Adjusted OR (95% CI)
No AKI	343/2,912 (11.8%)	Reference	66/983 (6.7%)	Reference
Stage 1	477/2,758 (17.3%)	1.42 (1.21–1.65)***	36/434 (8.3%)	1.01 (0.64–1.60)
Stage 2	926/3,974 (23.3%)	2.11 (1.83–2.43)***	87/669 (13.0%)	1.56 (1.08–2.25)**
Stage 3	579/1,174 (49.3%)	4.11 (3.40–4.97)***	26/127 (20.5%)	1.92 (1.07–3.44)**
Trend test	-	1.55 (1.46–1.64)***	-	1.25 (1.07–1.46)**

Values are presented as number of events/total number of patients (percentage). Odds ratios are adjusted for age, baseline SOFA score, baseline SAPS II score, baseline creatinine, and baseline lactate. Trend test evaluates AKI severity as an ordinal variable (0, 1, 2, 3). AKI, acute kidney injury; OR, odds ratio; CI, confidence interval. **p < 0.01, ***p < 0.001

The Reality cohort showed a similar pattern despite lower baseline delirium rates: 66 of 983 patients (6.7%) without AKI, 36 of 434 patients (8.3%) with Stage 1 AKI, 87 of 669 patients (13.0%) with Stage 2 AKI, and 26 of 127 patients (20.5%) with Stage 3 AKI. The adjusted odds ratios also increased with AKI severity: Stage 1 (OR 1.01, 95% CI 0.64–1.60, *p* = 0.96), Stage 2 (OR 1.56, 95% CI 1.08–2.25, *p* = 0.01), and Stage 3 (OR 1.92, 95% CI 1.07–3.44, *p* < 0.01) (Table 2). The trend test confirmed a significant biological gradient (OR 1.25 per stage increase, 95% CI 1.07–1.46, *p* < 0.001).

### Sensitivity analysis

To assess the temporal relationship between AKI timing and effect magnitude, we performed landmark analyses at 24-, 48-, 72-, and 96-hours post-ICU admission (Table S4). The association was strongest at the 24-hour landmark and gradually attenuated at later timepoints. Though the association between AKI severity and delirium rate remained consistent across all landmarks with all trend analysis at each timepoint reaching significance. Propensity score matching successfully balanced baseline characteristics between AKI and non-AKI groups at the 24-hour landmark. In MIMIC-IV, 8,642 matched pairs were created, and delirium rates remained significantly different (24.1% vs. 19.2%, *p* < 0.001 by McNemar’s test). In the Reality cohort, 1,230 matched pairs showed significant difference (12.1% vs. 7.4%, *p* < 0.001), confirming the robustness of the association. A sensitivity analysis restricted to patients that had daily CAM-ICU assessments yielded a cohort of 4064 patients for analysis. Crude delirium rates at the 24-hour landmark were 9.6% without AKI and 15.8% with AKI present, resulting in a risk difference of 6.2%. A logistic regression model was fitted and reached significance for the entire model (*p* < 0.001) and AKI at landmark remained a significant factor in contributing to delirium with an OR of 1.39 (CI 1.13–1.72). A detailed overview of all effect estimates is presented in Figure S4.

When the effect of decreased GFR is explored in the landmark analysis at 24 h significant differences for delirium proportions were seen in both cohorts. After dividing the patient cohorts by GFR and exclusion for missing data and early delirium, 2958 of 10,389 (28.5%) patients had low GFR at the landmark timepoint in the MIMIC cohort and 538 of 2213 (24.3%) in the Reality cohort. Both cohorts showed relevant differences in their delirium proportions. In MIMIC, 921 (31.1%) patients with a reduced GFR vs. 1165 patients with normal GFR (15.7%) showed delirium. OR was 2.43 (CI: 2.20–2.69). Logistic regression showed an OR of 1.3 (95% CI 1.19–1.34, *p* < 0.01). In the Reality cohort, 92 patients with exposure to low GFR showed delirium (17.1%) vs. 123 patients with normal GFR (7.3%). OR was 2.60 (CI: 91.6% − 241.6%). Logistic regression showed an OR of 1.15 without reaching significance thresholds (95% CI 0.97–1.36, *p* = 0.12).

In the Fine-Gray model, presence of AKI at the landmark timepoint showed a trend towards positive with an HR of 1.09 without reaching significance thresholds (CI 0.98–1.21, *p* = 0.12).

## Discussion

In this retrospective cohort study, we examined the association between AKI and delirium in critically ill patients using two independent databases collectively including over 18,000 patients. Our analysis revealed several key findings. First, AKI was associated with increased delirium risk in both cohorts. Second, we observed a direct association between severity of AKI and the rate of delirium, with adjusted odds ratios rising from 1.42 for Stage 1 to 4.11 for Stage 3 (trend test OR 1.55 per stage, *p* < 0.001). Finally, these findings remained consistent across multiple analytical approaches, including landmark analysis, propensity score matching, and were validated in two independent cohorts from different healthcare systems and patient populations.

Our study provides compelling evidence for an association between AKI and delirium in critically ill patients. Furthermore, in our study, this relationship appears to be independent of the severity of the underlying illness. The consistency of findings across two independent cohorts from different healthcare systems and patient populations, additionally strengthens the validity of our results. Given the high incidence of AKI in ICU settings, these findings are of considerable clinical relevance, suggesting that effective prevention of AKI could significantly modify the occurrence of delirium across ICU populations.

Our findings align with and extend previous research examining the relationship between AKI and delirium in critically ill patients. Several prior studies have identified AKI as a risk factor for delirium, though most were limited by smaller sample sizes, single-center designs, or methodological constraints [23, 28, 37]. Jäckel et al. [28] and Wan et al. [37] both demonstrated that AKI severity was associated with the occurrence of delirium, with higher AKI stages showing progressively greater risk. Specifically, both studies reported that patients with Stage 3 AKI experienced 13% to 28% higher delirium rates compared to those with Stage 1 AKI [28, 37]. Our results closely mirror these findings with differences of 32% in MIMIC-IV (49.3% vs. 17.3%) and 12.2% in the Reality cohort (20.5% vs. 8.3%) when comparing Stage 3 to stage 1 AKI patients. The consistency across multiple independent cohorts strengthens the evidence for a true biological gradient, although in the smaller cohort form the institutional database, confidence intervals between the groups overlap.

Sedation is a well-established and potentially modifiable risk factor for delirium in critically ill patients. Patients with AKI may experience altered pharmacokinetics and accumulation of sedative agents, thereby increasing delirium susceptibility. In our analyses, the association between AKI and delirium remained present after ad-justment for cumulative sedative exposure, suggesting that the observed relationship is not solely explained by differences in sedation practices. Nevertheless, sedation may be involved in the pathway between AKI and delirium, and residual confounding related to sedation depth or unmeasured ICU practices cannot be fully excluded. Our findings suggest that sedative exposure may partially mediate, rather than merely confound, the relationship between AKI and delirium. AKI may exacerbate the neurotoxic effects of sedatives and their metabolites through impaired renal clearance, systemic inflammation, and disruption of the blood–brain barrier, increasing central nervous system vulnerability. Accumulation of uremic toxins and altered blood–brain barrier permeability may further amplify neurocognitive dysfunction independent of sedative exposure. Thus, attenuation of the AKI–delirium association after sedative adjustment likely reflects overadjustment for variables on the causal pathway, rather than elimination of a spurious association, underscoring a biologically plausible link between renal dysfunction, neurotoxicity, and delirium risk. However, this does not establish causality, and further mechanistic studies are needed. Beyond sedation, polypharmacy may contribute to delirium risk in patients with AKI through accumulation of renally cleared drugs and adverse drug–drug interactions, particularly in the setting of impaired clearance. In addition, metabolic acidosis and uremic derangements accompanying AKI may adversely affect cerebral function and neurotransmission, providing a plausible biological link between renal dysfunction and acute brain dysfunction.

Siew et al. [23] provided important mechanistic insights by demonstrating that peak serum creatinine (SCr) levels were significantly associated with both delirium and coma in critically ill patients, suggesting that the severity of renal dysfunction rather than just its presence drives neurological complications. This aligns with our observation that Stage 3 AKI carried substantially higher risk (adjusted OR 4.11 in MIMIC-IV, OR 1.92 in Reality) compared to milder stages. The physiological plausibility of this relationship is supported by evidence that higher AKI stages are associated with elevated uremic toxin levels [38–40], which are known contributors to uremic encephalopathy and may increase delirium susceptibility.

An interesting finding in our study was the relatively modest effect of Stage 1 AKI, particularly in the Reality cohort where Stage 1 showed no significant association with delirium (OR 1.01, 95% CI 0.64–1.60, *p* = 0.96). This may reflect the heterogeneity of Stage 1 AKI diagnosis. As demonstrated previously, lower AKI stages are often diagnosed using urine output criteria alone, whereas higher stages are more frequently diagnosed by SCr or both KDIGO criteria [1, 41]. Importantly, AKI diagnosed by a single criterion has been associated with fewer complications than diagnoses based on both criteria [1], suggesting that Stage 1 AKI may represent a more heterogeneous group with varying clinical significance.

Our study addresses several important gaps in existing literature. First, unlike most prior studies that compared patients with and without delirium retrospectively [25, 26], we employed a landmark analysis to simulate a prospective investigation, that establishes temporal precedence of AKI before delirium onset, strengthening causal inference. Second, while previous studies typically focused on narrow patient populations – such as elderly patients [42], requiring mechanical ventilation [43], or single-center cohorts [28] – our analysis included over 18,000 patients from two independent databases representing diverse ICU populations (both surgical and medical) across different healthcare systems and time periods. Third, we controlled for a comprehensive set of confounders including validated illness severity scores (SOFA, SAPS II), baseline renal function and demographic parameters, demonstrating that the AKI-delirium association persists even after accounting for overall disease severity. Finally, our use of multiple complementary analytical approaches – with consistent results across methods provides further evidence of this association.

Given that AKI occurs in up to 50% of critically ill patients [1] and our observed absolute risk increases of 5–10%, approximately one additional case of delirium occurs for every 10–20 patients who develop AKI. Since preventive strategies have proven most effective in reducing delirium incidence and severity [44], identifying patients with AKI – particularly those with Stage 2 or 3 – as high-risk group could enable more targeted implementation of delirium prevention bundles and more efficient allocation of limited clinical resources. Furthermore, the biological gradient we observed suggests that interventions aimed at preventing AKI progression or mitigating its severity might also reduce delirium burden, though this hypothesis requires prospective validation.

Despite these strengths, our study has several important limitations that warrant consideration. First and most importantly, as a retrospective observational study, we cannot establish causality definitively. Second, our landmark analysis design, while methodologically sound for establishing temporal relationships, introduces inherent survivor bias by only including patients who survived beyond the landmark timepoint without developing early delirium. Alternative approaches, such as sampling at the time of first AKI occurrence and excluding patients with delirium before AKI, would introduce immortal time bias [45] and skew the AKI population toward healthier patients, resulting in questionable interpretability. We therefore chose the landmark approach as the most appropriate method to minimize time-dependent bias, accepting the trade-off of survivor bias. As a result, associations observed at later landmarks may be attenuated and should be interpreted alongside time-updated analyses. Third, delirium assessment variability between cohorts likely contributed to observed differences in event rates. The Reality cohort showed considerably lower delirium rates (9.7%) compared to MIMIC-IV (21.5%), suggesting systematic differences in detection practices or genuinely different patient populations. The lower delirium incidence observed in the Reality cohort likely reflects differences in assessment frequency and documentation practices rather than true biological differences. Less frequent delirium screening may result in underestimating the delirium rate. These differences should be considered when comparing absolute delirium rates across cohorts. However, a sensitivity analysis of a cohort restricted to patients that were assessed for delirium daily yielded similar results to the larger study population. We would also like to point out that while there are differences between the cohorts, the methods within the cohort for AKI and non-AKI patients are the same. Since we are not comparing patients from one cohort to another, we estimate similar bias for AKI and non-AKI patients within a single cohort. Using the lowest creatinine as baseline is not an optimal strategy for defining the baseline for AKI patients. A true baseline would be drawn from creatinine values from outpatient records with a stable kidney function and AKI can be misclassified by using this approach. Unfortunately, this data is often not available in ICU health record systems. While we assume that this bias should be evenly distribut-ed across the patient groups, we cannot safely rule out the possibility of asymmetric bias. Landmarking inherently excludes early delirium events and early deaths, potentially selecting a less severely ill subset of patients. As a result, it may attenuate or distort associations at later timepoints by conditioning the analysis on survival and event-free status up to the landmark. This selection may preferentially exclude patients with early AKI or delirium, potentially biasing effect estimates toward the null over longer follow-up.

Accounting for death as a competing risk attenuated the association between AKI and delirium. This suggests that patients with AKI may have a higher risk of early mortality, thereby reducing the opportunity to develop delirium. While the association between AKI and delirium did not reach statistical significance in the Fine–Gray model, the direction and magnitude of the effect were consistent across multiple analytic approaches. Given the competing risk of death and the observational nature of the study, we believe these results reflect clinical complexity rather than absence of an association.

This study demonstrates an association between AKI and subsequent development of delirium in critically ill patients across two large, independent cohorts, with Stage 3 AKI conferring up to four-fold increased odds of delirium even after adjustment for illness severity. While our findings show a similar trend across multiple analytical approaches and cohorts, the retrospective observational design, as well as the effect-attenuation by including death as a competing risk, precludes definitive causal conclusions between AKI and the development of AKI. AKI may act as a marker of overall systemic illness severity or organ crosstalk rather than a direct causal driver of delirium. Whether AKI directly causes delirium through mechanisms such as uremic toxin accumulation and neuroinflammation, or whether it primarily creates conditions that increase vulnerability to delirium, remains to be clarified.

However, the demonstrated association between advanced stages of AKI and a significantly increased risk of delirium is noticeable. Adding another aspect to the already extensive list of adverse outcomes linked to AKI progression [46, 47]. This highlights the critical and, in the literature, already well-established necessity of preventing such progression at the earliest possible phase. Consequently, the strict screening for AKI and timely application of care bundles is essential, particularly, but not limited, in patients with already manifest AKI [46, 48, 49].

Subgroup analysis of future prospective data might be able to discover patients at risk for targeted therapy in subsequent studies. Future prospective, multicentre studies with detailed mechanistic assessments are needed to establish causality and determine whether interventions targeting AKI prevention or treatment can reduce delirium burden and furthermore, improve outcomes in critically ill patients.

## Supplementary Information

Supplementary Material 1

## Data Availability

The MIMIC-IV database is licensed under the PhysioNet Credentialed Health Data License 1.5.0 and accessible via the PhysioNet platform. The institutional database is not publicly accessible due to local legal restrictions on patient data protection.

## Abbreviations

AKI: Acute kidney injury
CI: Confidence interval
CAM-ICU: Confusion Assessment Method for the Intensive Care Unit
EHR: Electronic health record
eGFR: Estimated glomerular filtration rate
GFR: Glomerular filtration rate
HR: Hazard ratio
ICU: Intensive care unit
KDIGO: Kidney Disease Improving Global Outcomes
MIMIC-IV: Medical Information Mart for Intensive Care IV
OR: Odds ratio
RRT: Renal replacement therapy
SAPS II: Simplified Acute Physiology Score II
SCr: Serum creatinine
SOFA: Sequential Organ Failure Assessment
