# Supplementary material for: Association between acute kidney injury and delirium in critically ill patients: a retrospective cohort study using two independent databases
Source: Crit Care. 2026 Mar 5;30:158. doi: 10.1186/s13054-026-05922-0 (PMC13063765; doi:10.1186/s13054-026-05922-0)
Supplement: Supplementary file 1 — Supplementary Material 1 [file 13054_2026_5922_MOESM1_ESM.docx]

Supplements

**Article Title**: Association between Acute Kidney Injury and Delirium in Critically Ill Patients: A Retrospective Cohort Study Using Two Independent Databases

**Journal Name**: Intensive Care Medicine

**Authors and Affiliations**: Christian Porschen, MD^1*^; Christian Strauß, MD^1*^; Sean M. Bagshaw, MD^2^; John A. Kellum^3^; G. Sven Meuth, MD/PhD^4^; Paul Brauckmann, MSc^5^; Nguyen Thi Ngoc Anh, MSc^6,7^; Michael Fujarski, MSc^5^; Keyvan Mahjoory, PhD^6,^ Carla Barbara Schwienhorst^1^ and Alexander Zarbock, MD^1^

## ^1^ Department of Anesthesiology, Intensive Care, and Pain Medicine, University Hospital Muenster, University of Muenster, Germany

^2^ Department of Critical Care Medicine, Faculty of Medicine and Dentistry, University of Alberta and Alberta Health Services, Edmonton, Alberta, Canada

^3^ Center for Critical Care Nephrology, Department of Critical Care Medicine, University of Pittsburgh, Pittsburgh, PA, USA

^4^ Department of Neurology, Medical Faculty, Heinrich-Heine University Düsseldorf, Germany

^5^ Institute for Medical Informatics, University of Muenster, Germany

## ^6^ Institute of Translational Psychiatry, University of Muenster, Germany

^7^ Institute of Mathematics, University of Muenster, Germany

^*^contributed equally and share first authorship

**Corresponding Author**:

Alexander Zarbock, MD

University Hospital Münster

Department of Anaesthesiology, Intensive Care and Pain Medicine

University Hospital Münster

48149 Münster

Germany

Phone: +49-251-83-47252

E-Mail: zarbock@uni-muenster.de

# Figures

**
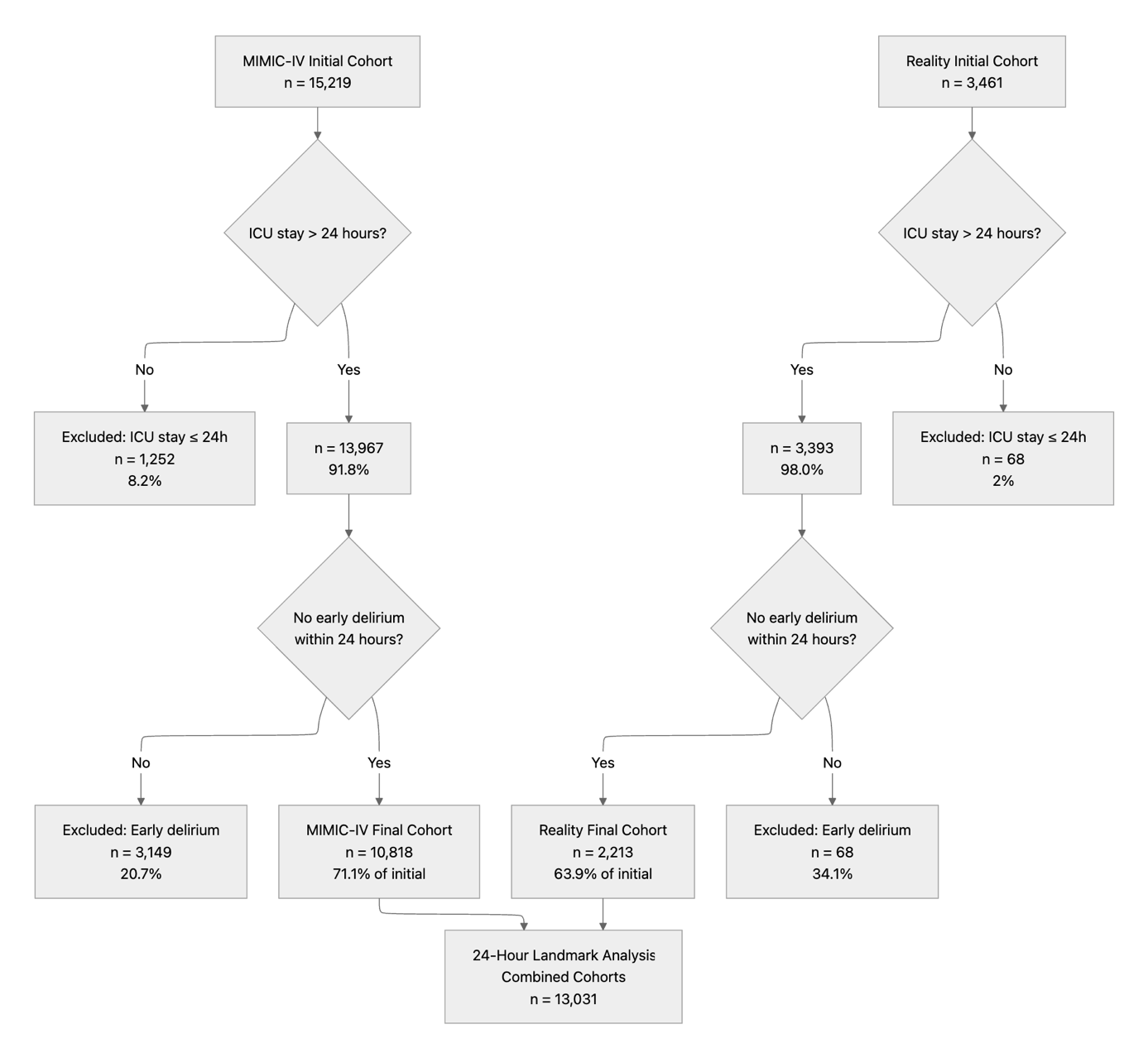
Supplemental Figure 1**: **Patient Selection Flowchart**. This flowchart shows patient selection for both cohorts in the 24-hour landmark analysis. The MIMIC-IV cohort started with 15,219 patients, with 1,252 (8.2%) excluded for ICU stays ≤24 hours and 3,148 (20.7%) excluded for early delirium, yielding 10,818 patients (71.1%) for final analysis. The Reality validation cohort included 3,461 patients, with no exclusions for short ICU stays and only 68 (2.0%) excluded for short ICU stay. Another 34.1% were excluded for early delirium, resulting in 2,213 patients (63.9%) for analysis. The combined final cohort comprised 13,031 patients eligible for 24-hour landmark analysis.


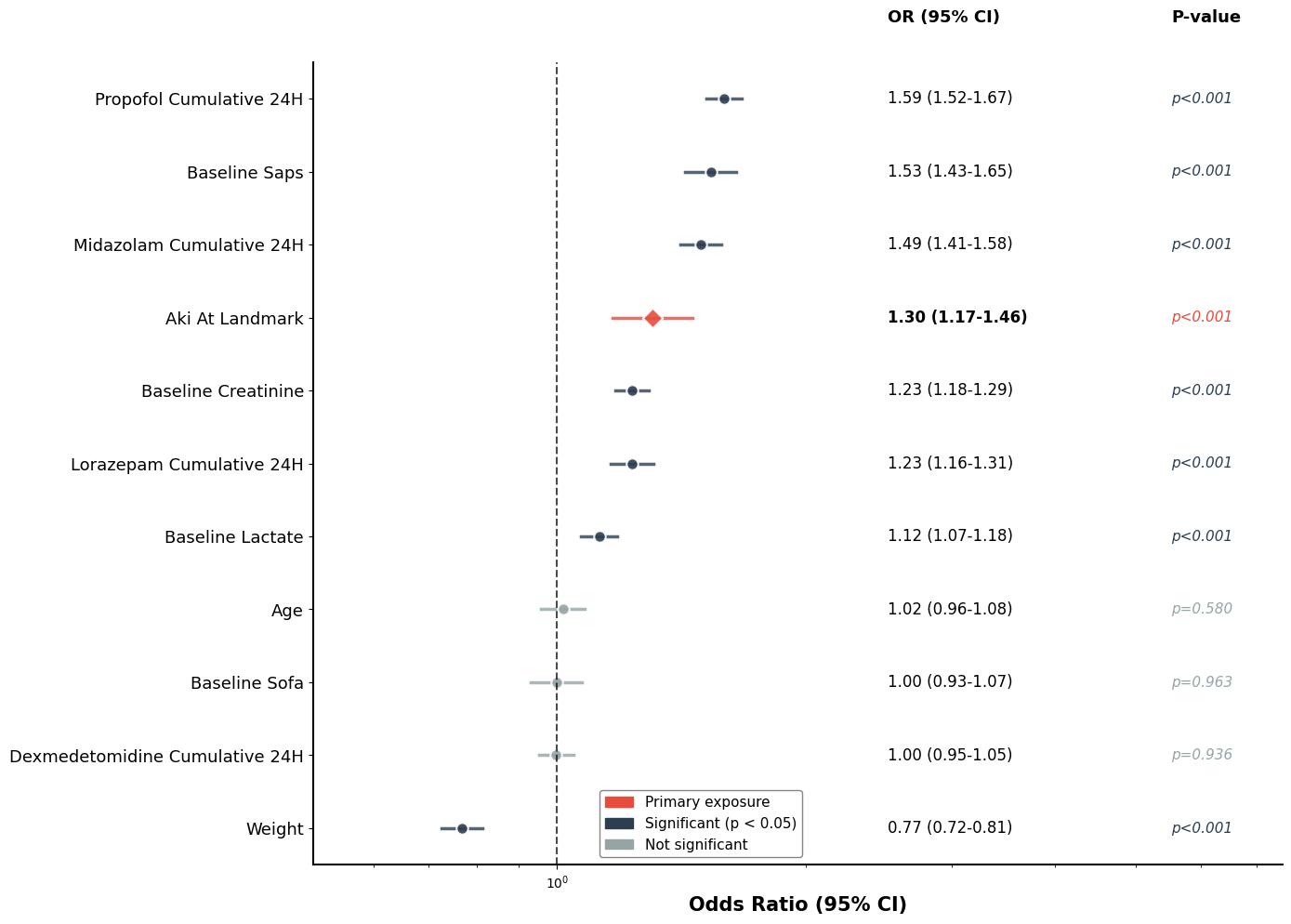


**Supplemental Figure 2: Risk Factors for Delirium in the MIMIC cohort**: Multivariable Logistic Regression. Adjusted odds ratios with 95% confidence intervals for delirium risk factors at 24-hour landmark. The model overall reached significance thresholds (p < 0.001). AKI presence was independently associated with delirium (OR 1.86, 95% CI 1.71-2.01, p<0.001) after adjusting for age, illness severity scores (SOFA, SAPS II), sedative exposure and baseline laboratory values. *p<0.05, **p<0.01, ***p<0.001.


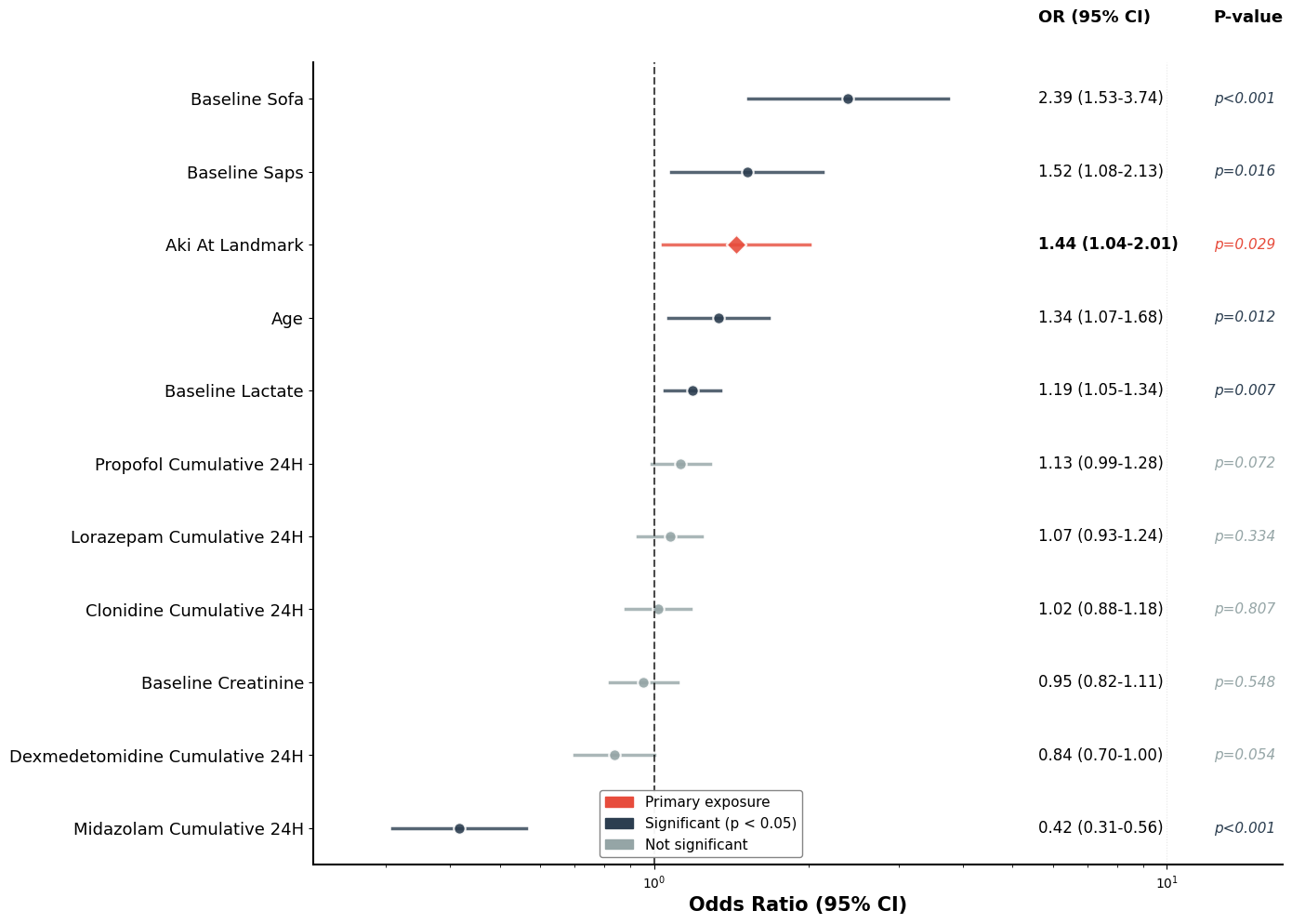


**Supplemental Figure 3**: **Risk Factors for Delirium in the Reality cohort**: Multivariable Logistic Regression. Adjusted odds ratios with 95% confidence intervals for delirium risk factors at 24-hour landmark. AKI presence was independently associated with delirium (OR 1.44, 95% CI 1.04-2.01, p=0.029) after adjusting for age, illness severity scores (SOFA, SAPS II), sedative exposure and baseline laboratory values. *p<0.05, **p<0.01, ***p<0.001.


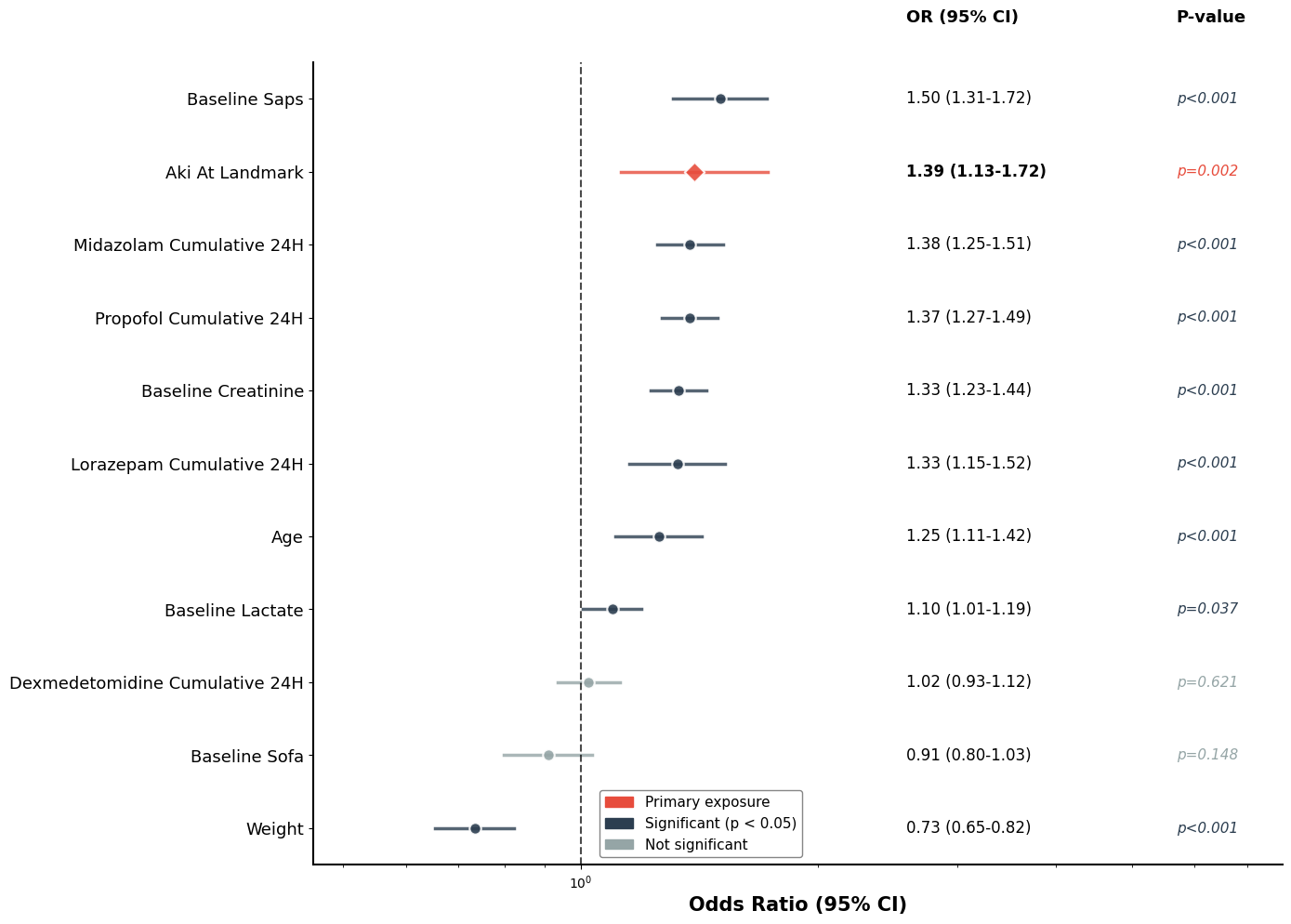


**Supplemental Figure 4**: **Risk Factors for Delirium in the Sensitivity Analysis, restricted to patients with daily CAM-ICU assessment only.** Adjusted odds ratios with 95% confidence intervals for delirium risk factors at 24-hour landmark. AKI presence was independently associated with delirium (OR 1.39, 95% CI 1.13-1.72, p=0.002) after adjusting for age, illness severity scores (SOFA, SAPS II), sedative exposure and baseline laboratory values. *p<0.05, **p<0.01, ***p<0.001.


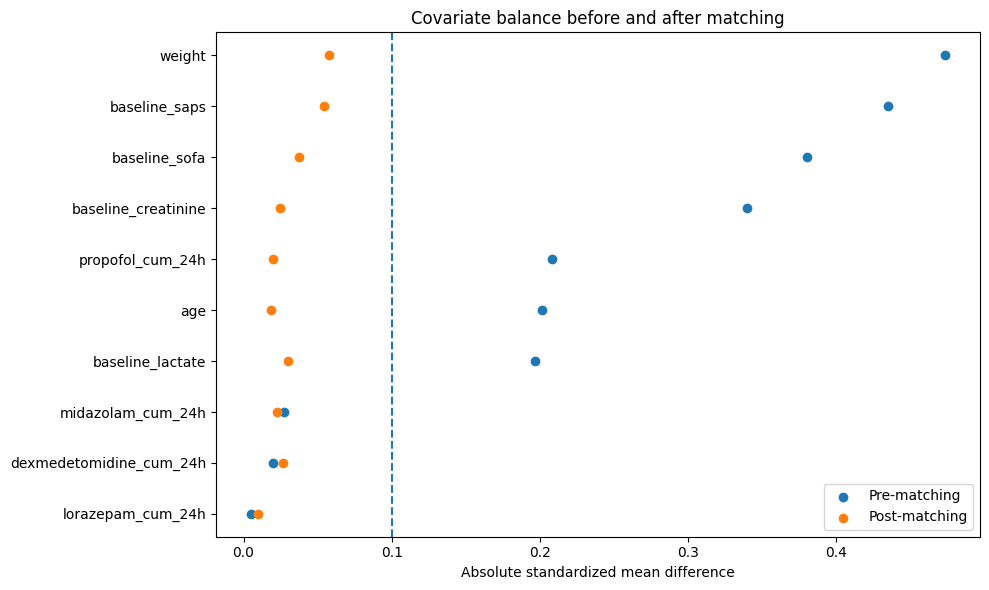


**Supplemental Figure 5: Covariate Balance in MIMIC before and after propensity score matching.** Love plot showing absolute standardized mean differences for baseline covariates before (blue) and after (orange) 1:1 propensity score matching. Post-matching, all covariates demonstrate improved balance, with standardized mean differences below the prespecified threshold of 0.1 (dashed line), indicating adequate covariate balance between groups.


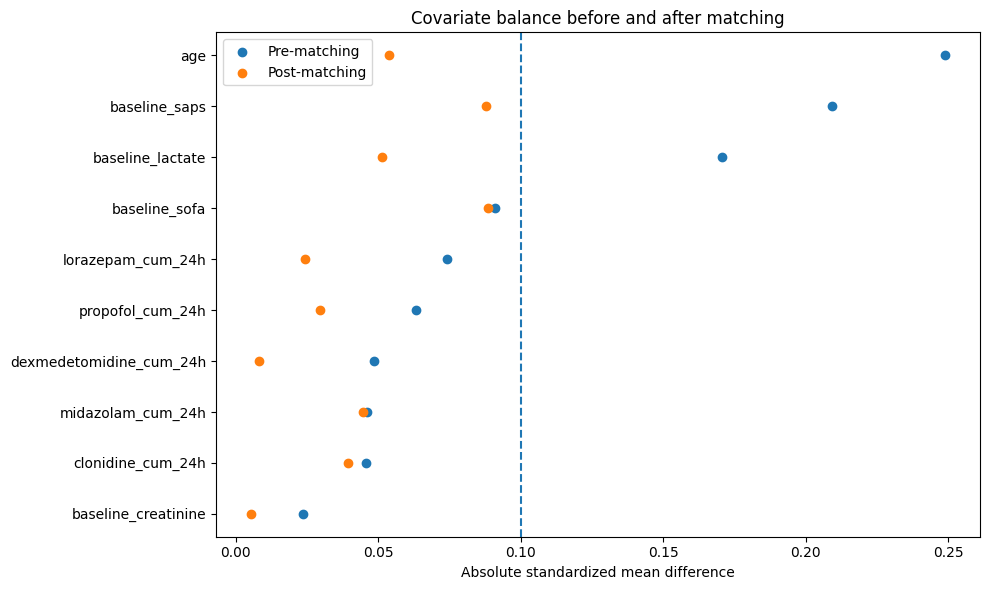


**Supplemental Figure 6**: **Covariate Balance in Reality Cohort before and after propensity score matching.** Love plot showing absolute standardized mean differences for baseline covariates before (blue) and after (orange) 1:1 propensity score matching. Post-matching, all covariates demonstrate improved balance, with standardized mean differences below the prespecified threshold of 0.1 (dashed line), indicating adequate covariate balance between groups.


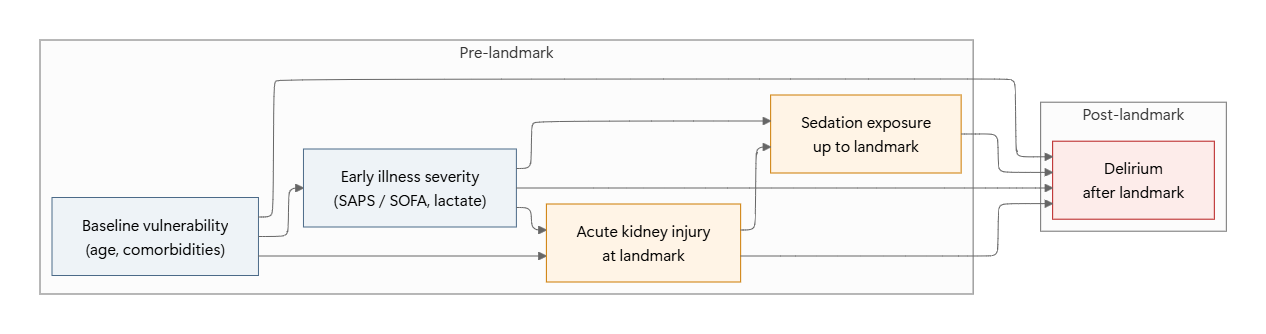


**Supplemental Figure 7:** **Directed acyclic graph illustrating the assumed causal structure between baseline vulnerability, early illness severity, sedation exposure, acute kidney injury, and subsequent delirium.** Baseline vulnerability (including age and comorbidities) influences early illness severity, the development of acute kidney injury at the landmark time, and the risk of delirium after the landmark. Early illness severity affects both acute kidney injury and cumulative sedation exposure up to the landmark, and also directly influences delirium risk. Acute kidney injury may increase delirium risk directly and indirectly through its impact on sedation exposure, reflecting potential pharmacokinetic and pharmacodynamic effects. Sedation exposure up to the landmark is modeled as a direct contributor to delirium occurring after the landmark. The graph reflects temporal ordering with exposures defined prior to the landmark and delirium assessed thereafter.

# Tables

| **Table 1: Baseline Characteristics****:** *Baseline demographics and clinical characteristics of patients in the Reality cohort (n=3,461) and MIMIC-IV cohort (n=15,219) included in the 24-hour landmark analysis. Values are presented as mean ± standard deviation for continuous variables and number (percentage) for categorical variables.* ***Abbreviations****: ICU, intensive care unit; RRT, renal replacement therapy; SAPS II, Simplified Acute Physiology Score II; SD, standard deviation; SOFA, Sequential Organ Failure Assessment.* | | | | | | |
| --- | --- | --- | --- | --- | --- | --- |
| **Characteristic** | **Reality Cohort (n=3,461)** | | | **MIMIC-IV Cohort (n=15,219)** | | |
|  | **Delirium (n=283)** | **No Delirium (n=3,178)** | **Total (n=3,461)** | **Delirium (n=5,650)** | **No Delirium (n=9,569)** | **Total (n=15,219)** |
| **Demographics and Baseline Clinical Characteristics** | | | | | | |
| Age, years (mean ± SD) | 70.8 ± 13.2 | 62.8 ± 15.2 | 63.5 ± 15.2 | 67.0 ± 15.7 | 66.0 ± 14.5 | 66.4 ± 15.0 |
| Male sex, n (%) | 182 (64.3) | 2,166 (68.2) | 2,348 (67.8) | 3,320 (58.8) | 6,014 (62.9) | 9,334 (61.3) |
| Weight, kg (mean ± SD) | 81.8 ± 19.5 | 83.9 ± 19.7 | 83.7 ± 19.7 | 84.7 ± 23.8 | 124.1 ± 3758.4 | 109.5 ± 2980.3 |
| SOFA score (mean ± SD) | 10.0 ± 3.4 | 6.9 ± 2.9 | 7.4 ± 3.1 | 5.5 ± 3.2 | 3.9 ± 2.6 | 4.5 ± 3.0 |
| SAPS II score (mean ± SD) | 47.6 ± 15.6 | 29.9 ± 11.9 | 31.4 ± 13.2 | 42.7 ± 14.6 | 34.6 ± 12.2 | 37.6 ± 13.7 |
| Baseline creatinine, mg/dL (mean ± SD) | 1.46 ± 1.06 | 1.09 ± 0.90 | 1.12 ± 0.92 | 1.60 ± 1.61 | 1.19 ± 1.17 | 1.34 ± 1.36 |
| Baseline lactate, mmol/L (mean ± SD) | 1.98 ± 1.43 | 1.37 ± 0.77 | 1.42 ± 0.86 | 2.43 ± 1.81 | 2.12 ± 1.13 | 2.24 ± 1.45 |
| RRT received, n (%) | 39 (13.8) | 57 (1.8) | 96 (2.8) | 825 (14.6) | 345 (3.6) | 1,170 (7.7) |
| Mechanical ventilation duration, hours (mean ± SD) | 90.6 ± 180.7 | 6.3 ± 31.7 | 13.2 ± 64.2 | 80.7 ± 138.0 | 11.6 ± 40.8 | 37.2 ± 96.1 |
| Length of ICU stay, days (mean ± SD) | 12.9 ± 13.5 | 3.0 ± 4.0 | 3.8 ± 6.1 | 7.5 ± 7.9 | 2.6 ± 2.6 | 4.4 ± 5.8 |

**Supplemental Table 2: Comorbidities and Baseline Medications in the Reality Cohort by Delirium Status:** Prevalence of pre-existing comorbidities and baseline medications (medications used prior to ICU admission) in patients from the Reality validation cohort stratified by delirium status. Values represent number (percentage) of patients with each comorbidity or receiving each medication class. Abbreviations: ACE, angiotensin-converting enzyme; NSAIDs, nonsteroidal anti-inflammatory drugs.

| **Characteristic** | **Delirium (n=283)** | **No Delirium (n=3,178)** | **Total (n=3,461)** |
| --- | --- | --- | --- |
| **Comorbidities, n (%)** | | | |
| **Diabetes Mellitus** | **94 (33.2)** | **724 (22.8)** | **818 (23.6)** |
| **Chronic kidney disease** | **63 (22.3)** | **474 (14.9)** | **537 (15.5)** |
| **Heart failure** | **123 (43.5)** | **1,050 (33.0)** | **1,173 (33.9)** |
| **Hypertension** | **171 (60.4)** | **1,518 (47.8)** | **1,689 (48.8)** |
| **Baseline Medications, n (%)** | | | |
| **ACE inhibitors** | **98 (34.6)** | **979 (30.8)** | **1,077 (31.1)** |
| **Angiotensin receptor blockers** | **66 (23.3)** | **731 (23.0)** | **797 (23.0)** |
| **Beta blockers** | **176 (62.2)** | **1,489 (46.9)** | **1,665 (48.1)** |
| **Diuretics** | **117 (41.3)** | **869 (27.3)** | **986 (28.5)** |
| **Insulin** | **41 (14.5)** | **275 (8.7)** | **316 (9.1)** |
| **Oral antidiabetics** | **64 (22.6)** | **551 (17.3)** | **615 (17.8)** |
| **NSAIDs** | **6 (2.1)** | **107 (3.4)** | **113 (3.3)** |
| **Antidepressants** | **37 (13.1)** | **303 (9.5)** | **340 (9.8)** |
| **Anticholinergics** | **22 (7.8)** | **294 (9.3)** | **316 (9.1)** |
| **Antiepileptics** | **29 (10.3)** | **226 (7.1)** | **255 (7.4)** |
| **Opioids** | **34 (12.0)** | **251 (7.9)** | **285 (8.2)** |
| **Benzodiazepines** | **29 (10.3)** | **363 (11.4)** | **392 (11.3)** |

| **Supplemental Table 3: Intra-ICU Sedative Use by Delirium Status:** Intra-ICU sedative medication use in patients from the Reality cohort and MIMIC-IV cohort. Values represent number (percentage) of patients who received each medication, along with mean ± standard deviation for the cumulative dose. Doses are reported as mean dose per administration. **Abbreviations:** ICU, intensive care unit; SD, standard deviation. | | | | | | | | | | |
| --- | --- | --- | --- | --- | --- | --- | --- | --- | --- | --- |
| **Sedative Agent** | **Reality Cohort (n=3,461)** | | | | | **MIMIC-IV Cohort (n=15,219)** | | | | |
|  | **Delirium (n=283)** | | **No Delirium (n=3,178)** | | **Total (n=3,461)** | **Delirium (n=5,650)** | | **No Delirium (n=9,569)** | | **Total (n=15,219)** |
|  | **n (%)** | **Dose** | **n (%)** | **Dose** |  | **n (%)** | **Dose** | **n (%)** | **Dose** |  |
| **Propofol (mg)** | **115 (40.6)** | **202.91 ± 149.0** | **770 (24.2)** | **141.5 ± 154.5** | **885 (25.6)** | **3,444 (61.0)** | **299.1 ± 194.7** | **5,257 (54.9)** | **259.4 ± 170.8** | **8,701 (57.2)** |
| **Dexmedetomidine (μg)** | **94 (33.2)** | **74.4 ± 83.6** | **1,056 (33.2)** | **100.3 ± 226.8** | **1,150 (33.2)** | **713 (12.6)** | **77.1 ± 62.9** | **933 (9.7)** | **52.4 ± 46.2** | **1,646 (10.8)** |
| **Midazolam (mg)** | **7 (2.5)** | **2.8 ± 1.7** | **8 (0.3)** | **37.2 ± 2.0** | **15 (0.4)** | **1,218 (21.6)** | **8.3 ± 12.4** | **564 (5.9)** | **5.7 ± 9.4  3.8 ± 3.5** | **1,782 (11.7)** |
| **Lorazepam (mg)** | **—** | | **—** | | **—** | **396 (7.0)** | **1.0 ± 0.7** | **317 (3.3)** | **0.7 ± 0.5** | **713 (4.7)** |
| **Clonidine (μg)** | **29 (10.2)** | **107.6 ± 56.8** | **87 (2.7)** | **125.1 ± 175.0** | **116 (3.4)** | **—** | | **—** | | **—** |

| **Diazepam (mg)** | **0 (0.0)** | **—** | **4 (0.1 )** | **6.5 ± 4.4** | **4 (0.1)** | **14 (0.2)** | **8.9 ± 4.9** | **14 (0.1)** | **5.3 ± 2.5** | **28 (0.2)** |
| --- | --- | --- | --- | --- | --- | --- | --- | --- | --- | --- |

**Supplemental Table 4**. **Sensitivity Analysis**: AKI Association and Gradient Severity-Response Relationship Across Multiple Landmark Timepoints: Association between AKI and delirium risk at different landmark timepoints in both cohorts. AKI OR represents the adjusted odds ratio for any AKI versus no AKI from multivariable logistic regression after the landmark time. Trend OR represents the odds ratio per one-stage increase in AKI severity (0→1→2→3) from gradient severity-response analysis, demonstrating the consistency of the gradient severity-response relationship across different temporal cutoffs. All models adjusted for age, baseline SOFA score, baseline SAPS II score, baseline creatinine, and baseline lactate. AKI, acute kidney injury; OR, odds ratio; CI, confidence interval.

| Landmark Time  (n) | MIMIC-IV | | | | Reality | | | |
| --- | --- | --- | --- | --- | --- | --- | --- | --- |
|  | LR OR (95% CI) | p-value | Trend OR (95% CI) | Trend p-value | LR OR (95% CI) | p-value | Trend OR (95% CI) | Trend p-value |
| 24 hours  (MIMIC: 10818 / Reality: 2213) | 1.16 (1.01-1.23) | 0.001 | 1.28 (1.22-1.34) | <0.001 | 1.20 (1.02-1.41) | 0.030 | 1.40 (1.15-1.70) | 0.001 |
| 48 hours  (MIMIC: 5591 / Reality: 1439) | 1.08 (1.00-1.16) | 0.05 | 1.55 (1.46 – 1.64 | <0.001 | 1.19 (0.97-1.47) | 0.10 | 1.43 (1.22 – 1.69) | <0.001 |
| 72 hours  (MIMIC: 3216 / Reality: 1001) | 0.99 (0.91-1.09) | 0.91 | 1.76 (1.66 – 1.86) | <0.001 | 1.22 (0.94-1.57) | 0.13 | 1.67 (1.40 – 2.00) | <0.001 |
| 96 hours  (MIMIC: 1939 / Reality: 701) | 0.88 (0.80-0.99) | 0.03 | 1.91 (1.80-2.03) | <0.001 | 1.26 (0.94-1.71) | 0.13 | 1.76 (1.47 – 2.12) | <0.001 |

**Supplemental Table 5: Adjusted Models**: Odds ratios (ORs) with 95% confidence intervals (CIs) from 24h landmark logistic regression models evaluating the association between acute kidney injury (AKI) at the landmark and post-landmark delirium in the MIMIC and Reality cohorts. Models are shown sequentially adjusting for AKI alone, baseline illness severity, and baseline severity plus cumulative sedative exposure.

| Cohort | Model | Variable | OR | 95% CI | P-value |
| --- | --- | --- | --- | --- | --- |
| Reality Cohort | AKI Only | AKI at landmark | 1.9467 | 1.436–2.639 | <0.001 |
|  | AKI + Severity Indicators | Age | 1.2266 | 0.989–1.521 | 0.0626 |
|  |  | Weight | 0.9378 | 0.794–1.107 | 0.4483 |
|  |  | Baseline creatinine | 0.9456 | 0.811–1.102 | 0.4752 |
|  |  | Baseline lactate | 1.1887 | 1.049–1.346 | 0.0066 |
|  |  | Baseline SOFA | 1.6845 | 1.294–2.193 | <0.001 |
|  |  | Baseline SAPS | 1.4999 | 1.130–1.991 | 0.0050 |
|  |  | AKI at landmark | 1.4771 | 1.055–2.067 | 0.0230 |
|  | AKI + Severity Indicators + Sedatives | Age | 1.3363 | 1.066–1.675 | 0.0119 |
|  |  | Baseline lactate | 1.1869 | 1.048–1.345 | 0.0071 |
|  |  | Baseline creatinine | 0.9544 | 0.820–1.111 | 0.5482 |
|  |  | Baseline SOFA | 2.3893 | 1.527–3.739 | <0.001 |
|  |  | Baseline SAPS | 1.5180 | 1.081–2.131 | 0.0158 |
|  |  | Propofol | 1.1276 | 0.989–1.285 | 0.0718 |
|  |  | Midazolam | 0.4168 | 0.309–0.563 | <0.001 |
|  |  | Dexmedetomidine | 0.8368 | 0.698–1.003 | 0.0538 |
|  |  | Clonidine | 1.0182 | 0.881–1.177 | 0.8068 |
|  |  | Lorazepam | 1.0738 | 0.929–1.241 | 0.3338 |
|  |  | AKI at landmark | 1.4449 | 1.038–2.011 | 0.0291 |
| MIMIC Cohort | AKI Only | AKI at landmark | 1.8480 | 1.675–2.039 | <0.001 |
|  |  |  |  |  |  |
|  | AKI + Severity Indicators | Age | 0.8622 | 0.815–0.912 | <0.001 |
|  |  | Weight | 0.8824 | 0.838–0.929 | <0.001 |
|  |  | Baseline creatinine | 1.1284 | 1.080–1.179 | <0.001 |
|  |  | Baseline lactate | 1.1221 | 1.069–1.178 | <0.001 |
|  |  | Baseline SOFA | 1.1469 | 1.073–1.225 | <0.001 |
|  |  | Baseline SAPS | 1.6340 | 1.527–1.749 | <0.001 |
|  |  | AKI at landmark | 1.3551 | 1.216–1.510 | <0.001 |
|  |  | | | | |
|  | AKI + Severity Indicators + Sedatives | Age | 1.0172 | 0.958–1.080 | 0.5799 |
|  |  | Weight | 0.7675 | 0.725–0.813 | <0.001 |
|  |  | Baseline creatinine | 1.2325 | 1.176–1.292 | <0.001 |
|  |  | Baseline lactate | 1.1245 | 1.070–1.182 | <0.001 |
|  |  | Baseline SOFA | 0.9983 | 0.930–1.071 | 0.9628 |
|  |  | Baseline SAPS | 1.5347 | 1.429–1.648 | <0.001 |
|  |  | Propofol | 1.5924 | 1.516–1.673 | <0.001 |
|  |  | Midazolam | 1.4934 | 1.410–1.581 | <0.001 |
|  |  | Dexmedetomidine | 0.9980 | 0.952–1.047 | 0.9357 |
|  |  | Lorazepam | 1.2320 | 1.160–1.309 | <0.001 |
|  |  | AKI at landmark | 1.3050 | 1.167–1.459 | <0.001 |
